# Supplementary material for: Endocrine Mucin-Producing Sweat Gland Carcinoma (EMPSGC) in a Dog: Immunohistochemical Characterization
Source: Animals (Basel). 2024 Dec 17;14(24):3637. doi: 10.3390/ani14243637 (PMC11672491; doi:10.3390/ani14243637)
Supplement: Supplementary file 1 [file animals-14-03637-s001.zip › animals-3297510-supplementary.pdf]

animals

MDPI

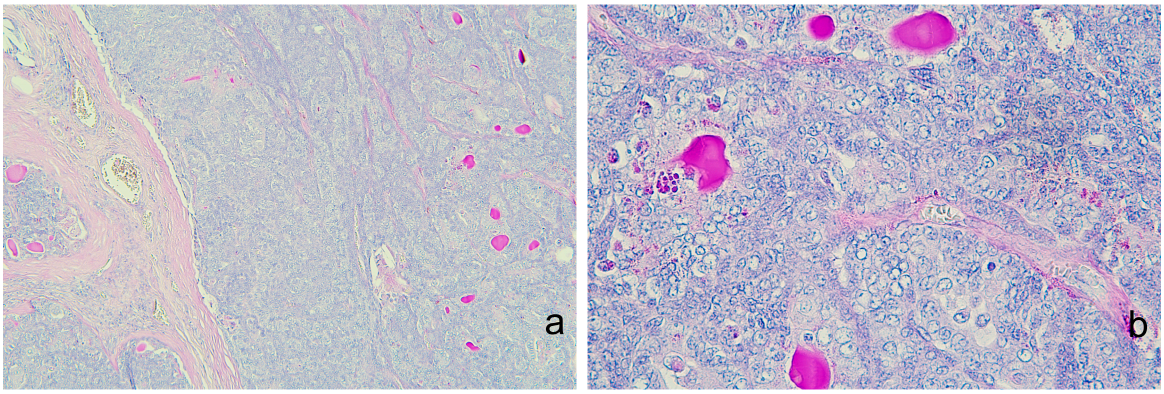

Figure S1. Periodic Acid-Schiff (PAS) Staining of Endocrine Mucin-Producing Sweat Gland Carcinoma (EMPSGC). (A) The tumor demonstrates prominent mucin accumulation, indicated by the intense magenta staining within the tumor cells and extracellular matrix. The mucin appears as bright, magenta-colored areas, characteristic of PAS-positive material. Magnification = X100. (B) High magnification reveals distinct mucin-filled spaces, confirming the presence of abundant mucin in the tumor cells and interstitial areas. The mucin appears more defined at this magnification, further highlighting the PAS-positive staining pattern. Magnification = X400.
